# Desipramine enhances the stability of atherosclerotic plaque in rabbits monitored with molecular imaging

**DOI:** 10.1371/journal.pone.0283612

**Published:** 2023-03-30

**Authors:** Min Zhao, Baiyang You, Xiaole Wang, Jin Huang, Ming Zhou, Ruizheng Shi, Guogang Zhang

**Affiliations:** 1 Department of Nuclear Medicine, Xiangya Hospital of Central South University, Changsha, China; 2 National Clinical Research Center for Geriatric Disorders, Xiangya Hospital, Changsha, Hunan, China; 3 Division of Cardiac Rehabilitation, Department of Physical Medicine & Rehabilitation of Xiangya Hospital of Central South University, Changsha, China; 4 Department of Pediatrics, Xiangya Hospital of Central South University, Changsha, China; 5 Department of Cardiovascular Medicine, Xiangya Hospital of Central South University, Changsha, China; 6 Department of Cardiovascular Medicine, The Third Xiangya Hospital of Central South University, Changsha, China; Yanbian University Hospital, CHINA

## Abstract

Acid sphingomyelinase (ASM) promotes atherogenesis and acute cardiovascular events. We previously demonstrated ASM inhibitor desipramine attenuated oxidized-LDL-induced macrophage apoptosis in vitro. Here, we aim to determine whether ASM-mediated apoptosis in plaque improves stability in vivo. In this study, rabbits with abdominal aorta balloon injury and a 12-week high-cholesterol diet (HCD) were used to simulate an atherosclerotic plaque model. Atherosclerotic rabbits received oral administration of saline (Control group), atorvastatin (Ator group), or desipramine (DES group). ASM activity and ceramide level were measured by ultra-performance liquid chromatography (UPLC). Plaque morphology was assessed by histochemistry and immunohistochemistry. Apoptosis was evaluated by SPECT/CT imaging of 99mTc-duramycin uptake and TUNEL. We found that increasing ASM activity and ceramide level in atherosclerotic rabbits was abated by additional atorvastatin and desipramine treatment. Meanwhile, the DES and Ator groups were similar in plaque stability, with smaller plaque size, areas of macrophages, higher smooth muscle cell content, and decreased apoptosis and matrix metalloproteinase (MMP) activities relative to the Control group. 99mTc-duramycin uptake of rabbit aorta was significantly higher in Control than in the Normal group, while it was reduced by desipramine and atorvastatin administration. Moreover, the uptake of 99mTc-duramycin positively correlated with apoptotic cell number, macrophage infiltration, and plaque instability. The present study demonstrated that desipramine exerted plaque-stabilizing effects partially by suppressing apoptosis and MMP activity in a rabbit model. And ^99^mTc-duramycin SPECT/CT imaging allowed noninvasively monitoring of atherosclerotic disease and evaluation of anti-atherosclerotic therapy.

## Introduction

Atherosclerosis is the primary cause of cardiovascular diseases, which are the major cause of morbidity and mortality worldwide. Most agents were treated for atherosclerosis by reducing plasma low-density lipoproteins (LDLs), which substantially reduces major adverse cardiovascular events (MACE) [1–3]. The most effective drugs to date are statins. However, statins may not be tolerated well by all patients at very high doses. Apart from cholesterol, sphingolipids were also widely studied in atherosclerotic plaque progression [4, 5]. Among sphingolipids, ceramide is the most bioactive lipid that plays a central role in cell membrane integrity, cellular stress responses, inflammatory signaling, and apoptosis [6]. Recent clinical trials have shown that circulating ceramide, as a new biomarker, was strongly associated with cardiovascular events, such as myocardial infarction and stroke [7, 8], implying that ceramide-reducing intervention might be a potential novel therapeutic approach for atherosclerosis.

Ceramide can be generated by various enzymes: sphingomyelinase, de novo synthesis, and salvage pathways [9]. Among them, the acid sphingomyelinase (ASM), which catalyzes the conversion of sphingomyelin into ceramide, is considered a key enzyme of cellular sphingolipid metabolism, implicated in lipoprotein retention, foam cell formation, and inflammation by stress agents such as oxidized-LDL, inflammatory cytokines, and oxidative stress [10, 11]. As a result of the differential post-translational processing, ASM gives rise to two distinct enzymes: secreted form (S-ASM) and lysosomal form (L-ASM), which might differ in their contribution to specific pathologic states. S-ASM was increased in murine models of atherosclerosis and patients with coronary heart disease [12–14]. In vitro treatment of LDL with human recombinant S-ASM induced the formation of lesion-like LDL aggregates; while inhibition of ASM exhibited both a decrease in lesion development and reduced arterial trapping of atherogenic lipoproteins in ApoE^−/−^ or LDLR^−/−^ mice [15]. Indeed, functional inhibitors of ASM with tricyclic antidepressants have emerged as promising drugs with broad therapeutic potential for bacterial infections and cancer [16, 17]. The in vitro studies revealed that desipramine, an FDA-approved tricyclic antidepressant that functionally inhibits ASM, prevented ox-LDL-induced macrophage apoptosis and matrix metalloproteinase (MMP) induction [18, 19]. Yet, its anti-atherosclerosis potential in vivo remains largely unexplored.

Phosphatidylethanolamine (PE) is more widely expressed in apoptotic cells compared with phosphatidylserine (PS) and can be targeted with radiolabeled duralumin for molecular imaging using single-photon emission computed tomography (SPECT). Recently, a novel PE-binding imaging probe, ^99m^Tc-duramycin, has been proven to be feasible in detecting atherosclerotic apoptosis in various experimental models of atherosclerosis [20–22]. It has several advantages including a better target-to-background ratio and lower non-target organ radiation burden than the PS-binding imaging tracer, such as ^99m^Tc-annexin A5 or ^99m^Tc-annexin V [23]. However, it has not been investigated whether ^99m^Tc-duramycin imaging can be used to monitor anti-atherosclerosis treatment effectiveness.

Accordingly, this study aimed to assess the effect of desipramine intake on experimentally induced atherosclerosis in rabbits, by comparing it with atorvastatin intake [24]. Additionally, we further evaluate the value of targeting imaging with duramycin to monitor disease progression and therapy efficacy.

## Methods

### Ethics statement

All animal protocols were approved by the Animal Care and Use Committee at Xiangya Hospital of Central South University, China (SYXK 2020–0019, Changsha). All methods mentioned in this manuscript were conducted by the guidelines for the use of live animals of the National Institute of Health to minimize the number of animals used and their suffering, which is also in compliance with relevant ARRIVE guidelines.

### Experimental animals and induction of atherosclerosis

Thirty-two male New Zealand white rabbits (2.5–3.5 kg, aged 12 weeks) were purchased from Tianqin Laboratories Inc (Changsha, China). Atherosclerosis was induced in rabbits by feeding with a 1% cholesterol diet custom mixed with 4% porcine oil and 10% powdered egg yolk. Two weeks after initiation of the high-cholesterol diet (HCD), the balloon de-endothelialization of the abdominal aorta through a balloon injury for induction of atherosclerotic lesions as previously reported [25]; Briefly, after anesthesia, the heparin sodium was administrated (i.v., 200 U/kg), and the femoral artery of right leg exposed and separated. Then, scissored it and inserted the PTCA balloon (3.5 × 15 mm, Boston Scientific, US) along the catheter to the abdominal aorta (20 cm). Next, inflated the balloon to 12 atm (1216 kPA) pressure, and withdrew the balloon slowly to the iliac artery (5–7 cm) twice. Finally, evacuated the balloon, withdrew all of the balloon and catheter, ligated the femoral artery, and close the wound. In this procedure, 3% pentobarbital (i.v. 30mg/kg) was used for anesthesia, and 60mg/kg (i.v.) pentobarbital was used for euthanasia. HCD feeding was then continued for 10 more weeks.

### Animal grouping

**Fig 1:**
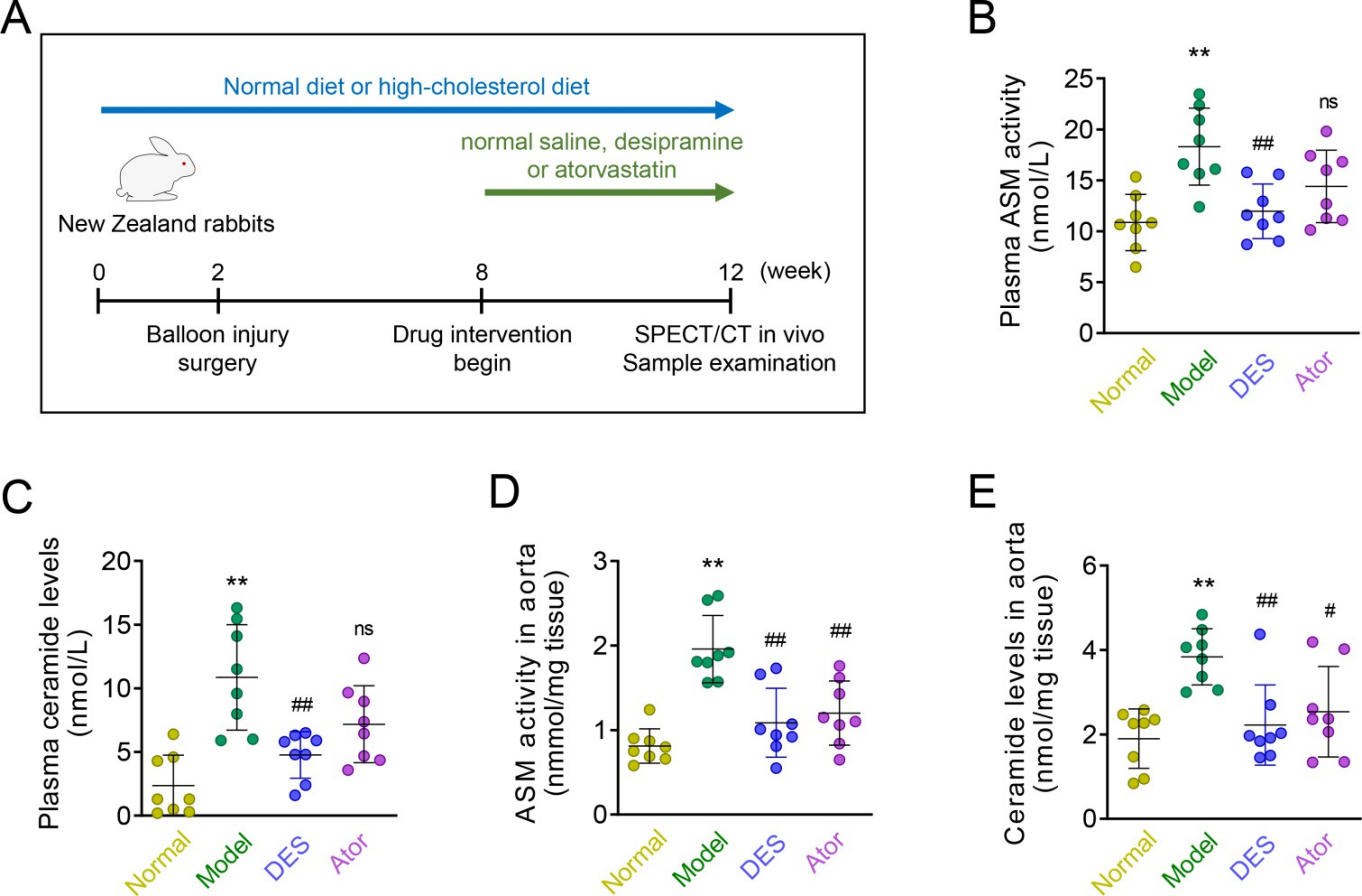
Desipramine and atorvastatin inhibited ASM and ceramide. **(A)** New Zealand white rabbits were randomly divided into four groups: Rabbits were fed on normal chow diet served as normal controls (Normal); Abdominal aorta balloon injury surgery was performed on high-cholesterol diet feeding rabbits after 2 weeks, then normal saline (Model), desipramine (DES), and atorvastatin (Ator) were administrated by gavage from 8th to 12th week. Finally, SPECT/CT and sample examination was performed. **(B, C)** Blood was obtained, and ASM activities and ceramide levels in plasma were determined by UPLC; **(D, E)** ASM activities and ceramide levels in aorta tissue were assessed by UPLC. Data are expressed as Mean ± SD, n = 8; *, ** represent *P* <0.05, *P* <0.01 in comparison with Normal; #, ## represent *P* <0.05, *P* <0.01 in comparison with Model, ns represents no-significance in comparison with Model.

At week 8, The normal control group rabbits (n = 8) were fed on normal chow and received saline orally every day for 4 weeks. The experimental atherosclerotic rabbits have distributed among 3 groups with 8 rabbits in each group: the atherosclerosis model group received saline orally every day for 4 weeks; the desipramine group received desipramine (Sigma, MO, USA) at a dose of 4 mg/kg/day for 4 weeks; atorvastatin group received atorvastatin (Pfizer, NY, USA) at a dose of 2.5 mg/kg/day for 4 weeks. The calculation of the drug dose was based on the body surface area formula for therapeutic human dosing (desipramine of 200 mg/day and atorvastatin of 40 mg/day). Drugs were dissolved in saline and administrated by oral gavage. An equal volume of saline was administered in the normal and model groups. The study protocol was summarized in **Fig 1A**. The investigators who performed followed examinations such as lipid profile, ASM activities, and imaging, are blinded to the animal group.

### Lipid profile assays

Blood samples were collected from the rabbit ear margin vein and divided equally into two different disposable non-coagulant heparin tubes, one for serum and another for plasma. Serum and plasma were isolated by centrifugation at 4000 rpm for 10 minutes at room temperature. The supernatants were collected and stored at -80°C. Serum levels of total cholesterol (TC), triglycerides (TG), and low-density lipoprotein cholesterol (LDL) were measured by commercial enzymatic test kits according to the manufacturer’s instructions using an automatic analyzer (Beckman, IN, USA).

### ASM activities and ceramide assays

Plasma and vascular ASM activity and ceramide levels were measured by the UPLC method as in our previous report [26]. For the ASM activity assay, the hydrolytic product was detected and quantified by the UPLC system (Acquity UPLC H-class, Waters, Milford, MA, USA). In this system, a reversed-phase column (Acquity BEH Amide, 2.1 × 50 mm, 1.7 μm, Waters, USA) was used. For ceramide assay, lipids extraction and ceramide quantification were performed as previously described [26], and a reversed-phase column (Acquity BEH Shield RP18, 2.1 × 50 mm, 1.7 μm, Waters, USA) was been used in this experiment.

### Imaging reagents and radiolabeling

A modified single-step kit protocol based on the method described by Zhao was used [23]. Briefly, duramycin (Sigma) was conjugated with hydrazinonicotinic acid (HYNIC; Solulink, CA, USA) at a molar ratio of 20:1 in anhydrous dimethylformamide. HYNIC-conjugated duramycin was purified by C_18_ reversed-phase ultra-performance liquid chromatography (UPLC) at a flow rate of 4 mL/min at room temperature. For radiolabeling with ^99m^Tc, 100 μg HYNIC-conjugated duramycin was added to 100 mg tricine (Sigma), 32 mg trisodium triphenylphosphine-3,3,3-trisulfonate (Lianshuo, Shanghai, China), and 21 μg SnCl_2_ (Sigma) in a volume of 2 mL. Aliquots of 500 μL were freeze-dried overnight. The reaction vial was mixed with approximately 740 MBq of ^99m^Tc-sodium pertechnetate (Atom High Tech, Beijing, China) in 500 μL of saline and then heated to 80°C for 20 min. The radiochemical purity was greater than 98%, as determined by instant thin-layer chromatography.

### In vivo imaging and ex vivo assessment

Three hours after injection of ^99m^Tc-duramycin (74–111 MBq), rabbits were sedated and anesthetized with ketamine (20 mg/kg) and xylazine (5 mg/kg) and then placed in a supine position in a clinical dual-head SPECT/CT scanner (Precedence16, Philips Healthcare, Cleveland, OH, USA). SPECT acquisition covering from the aortic arch to the bifurcation was performed with 32 projections (10 s per projection) on the 140 keV photopeak of ^99m^Tc with 15% windows, using a low-energy higher solution collimator. SPECT images were reconstructed into a matrix size of 128 × 128 pixels and a pixel size of 1.46 × 1.46 mm using the Astonish algorithm. A CT scan (100 mA, 130 kV, 1 mm-slice thickness) was acquired for subsequent SPECT/CT fusion. After in vivo imaging, animals were sacrificed with an overdose of pentobarbital, blood was drawn via cardiac puncture, and the aorta from the aortic arch to the iliac artery bifurcation was harvested and segmented at 1-cm intervals. Each segment was weighed and radioactivity concentration (decay corrected) was measured in an automatic gamma well counter (Zhongjia-1200 γ-counter, Zhongjia Guangdian Co. China). The results were expressed as the percentage of injected dose per gram of tissue (%ID/g). The harvested aortas were further processed for histological studies.

### Histological assessment

The radioactivity concentration of the upper abdominal aorta segment is the highest, which was selected for the experiment. This segment was separated into several portions, and one of them was fixed overnight in 4% paraformaldehyde solution, embedded in paraffin, and cut into 4-μm serial cross-sections. One section was used for hematoxylin and eosin (H&E) staining and the adjacent sections were used for immunohistochemistry. Immunostaining was assessed for macrophages, smooth muscle cells (SMCs), MMP-2, and MMP-9, using anti-rabbit RAM-11 (Dako, Carpinteria, CA, USA), α-SMA (Abcam, Cambridge, UK), MMP-2 (Sigma-Aldrich, MA, USA), MMP-9 (Sigma-Aldrich, MA, USA), respectively. After staining, the sections were scanned with a microscope (Leica DMI 3000B, Wetzlar, Germany). The relative areas of each tissue component were expressed as a percentage of positively stained area divided by the intima area (%) using Image-Pro Plus 6.0 software (Media Cybernetics, MD, USA). 6 sections in each group were used for analysis.

### TUNEL staining

Paraffin-embedded aorta slices were stained by TUNEL assay using an *in situ* cell death detection kit-POD (Roche Diagnostics, Indianapolis, IN, USA) to evaluate apoptosis, as the previously described procedure [27]. The slides were photographed by fluorescence microscopy. In total, 200 cells under five high-power fields (×200) from 10 cross-sections of each rabbit were imaged and counted.

### Gelatin zymography

Evaluating the presence of both activated and pro-forms of MMP-2 and MMP-9 has been described previously [28]. The MMP zymography assay kit was purchased from Applygen Technologies, Beijing, China. Briefly, frozen abdominal aortas were weighed and homogenized. The total protein was normalized using a Lowry protein assay kit (Bio-Rad) and resolved by 11% sodium dodecyl sulfate (SDS)-polyacrylamide gel electrophoresis (PAGE) copolymerized with gelatin (1 mg/mL). Gels were washed in 2.5% Triton X-100 to remove SDS and allow renaturation of MMPs, transferred to developing buffer (50 mM Tris pH 7.5, 5 mM CaCl_2_, 1 μM ZnCl_2_), and incubated overnight at 37°C under continuous shaking. The activities of MMP-2 and MMP-9 were detected with a ChemiDoc MP Imaging system (Bio-Rad). The relative expression levels of proteins were normalized against the normal group.

### Statistical analysis

All statistical analyses were performed using SPSS 26.0 (IBM, Somers, NY, USA). Quantitative data are expressed as mean ± standard deviation (SD). Differences were analyzed by one-way analysis of variance (ANOVA) followed by the Bonferroni test for 4 groups. The positivity percentage of histopathology for each cross-section was correlated with the cross-section’s corresponding ^99m^Tc-duramycin uptake by using linear regression. The results were considered statistically significant at *P*< 0.05.

## Results

### Effects of desipramine and atorvastatin on lipid profiles

The atherosclerotic rabbits were treated with saline, desipramine, or atorvastatin, and the detailed study protocol was summarized in **Fig 1A**. After 12 weeks of HCD feeding, plasma and vascular ASM activities and ceramide levels were significantly increased relative to animals on normal chow (all *P*<0.01). Concurrent treatment with desipramine and atorvastatin could attenuate HCD-induced ASM and ceramide accumulation in aortas, while only desipramine significantly suppressed ASM and ceramide increasing in plasma (**Fig 1B–1E**). Despite differences in diet, final body weights did not differ among the four groups at week 12 (**S1 Table**). As for serum lipid profiles, HCD-fed rabbits showed markedly elevated serum levels of TG, TC, and LDL when compared with the normal controls. Indeed, atorvastatin treatment lowered the serum TG, TC, and LDL, whereas desipramine administration did not show any lipid-lowering effect (**S1 Table**).

### Effects of desipramine and atorvastatin on morphological stability

**Fig 2:**
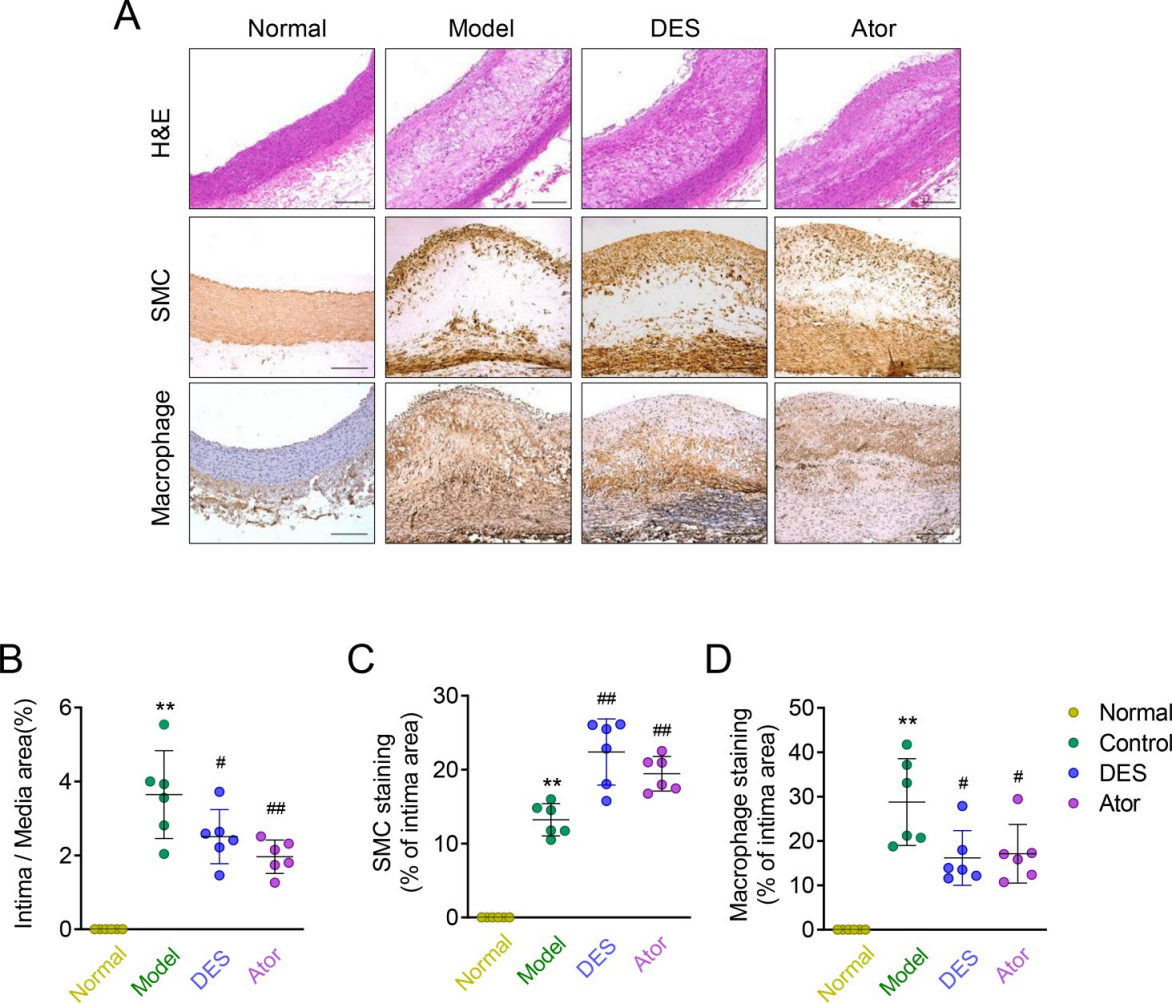
Desipramine and atorvastatin enhances atherosclerotic plaque stability. **(A)** Normal and atherosclerotic rabbits were treated with saline (Model), desipramine (DES), or atorvastatin (Ator), representative histological and Immunohistological staining of abdominal aortic plaque components including alpha smooth muscle actin (α-SMA) staining of SMCs, and RAM-11 staining of macrophages. Scale bar = 250 μm; **(B)** The ratio of intima/media area in each rabbit was analyzed; **(C, D)** Quantitative analysis of SMCs and macrophages in 4 groups. Data are expressed as Mean ± SD, n = 6; *, ** represent *P* <0.05, *P* <0.01 in comparison with Normal; #, ## represent *P* <0.05, *P* <0.01 in comparison with Model.

As shown in the representative histological images and quantitative data in **Fig 2**, lesion in the model group was evident, as represented by the markedly thickened intima and accumulation of macrophages. The administration of desipramine and atorvastatin for 4 weeks reduced the thickness ratio of the intima to media by ~40% or 50%, when compared with the model controls. In addition, there was a decrease in macrophage populations and an increase in SMC content in the desipramine and atorvastatin-treated group compared to the model controls.

### Effects of desipramine and atorvastatin on vascular MMPs activities and apoptosis

**Fig 3:**
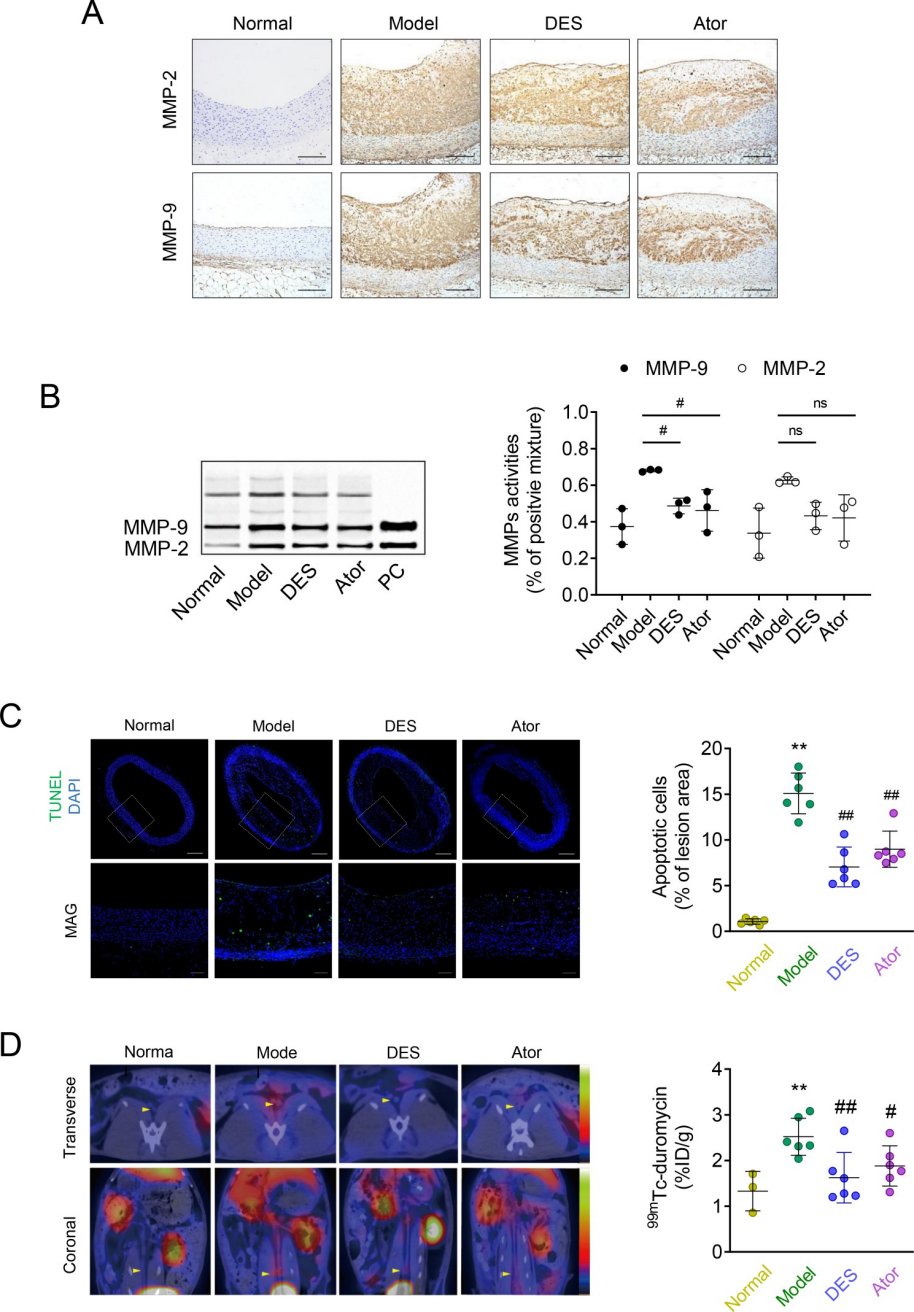
Desipramine and atorvastatin inhibit MMPs and apoptosis in atherosclerotic plaques. **(A)** Immunohistological staining and positive area analysis of MMP-2 and MMP-9 in abdominal aortas in 4 groups. Scale bar = 250 μm; **(B)** Zymographic analysis of MMP-2 and MMP-9 activities, the densitometry of each band was determined by ImageJ software and calculated as the percentage of the positive control (PC) on each gel. **(C)** Representative micrographs and quantification of TUNEL-positive (green) cells in aortic atherosclerotic plaques. Scale bar = 50 mm; **(D)** *In vivo* imaging of abdominal aorta (arrowheads) with ^99m^Tc-duramycin SPECT/CT and measurement of radioactive ^99m^Tc-duramycin uptake in aortic; n = 3 and 6. *, ** represent *P* <0.05, *P* <0.01 in comparison with Normal; #, ## represent *P* <0.05, *P* <0.01 in comparison with Model, ns represents no-significance in comparison with Model.

Plaque stability was partially determined by MMPs. The histopathological examination revealed marked MMP-2 and MMP-9 staining in aortic neointimal lesions in the model controls. However, in the desipramine and atorvastatin-treated group, MMP-2 and MMP-9 staining areas were smaller than that in the model controls (**Fig 3A**). SDS-PAGE zymography results showed that active MMP-2 and active MMP-9 gelatinolytic expressions in the model control group were higher than those in the normal control group, while both desipramine and atorvastatin treatment significantly decreased the activity of MMP-9 compared with the model controls. The activity of MMP-2 also shows a similar trend, but no statistical significance was observed (**Fig 3B**). To directly assess apoptosis in situ, lesions were stained by the TUNEL method to detect apoptotic cells. The quantification data showed ~15% TUNEL-positive nuclei in the model control lesions (**Fig 3C**). Most importantly, lesions from desipramine and atorvastatin groups had approximately one-third and half, respectively, fewer TUNEL-positive nuclei than lesions from the rabbit models. Similar data were obtained using ^99m^Tc-duramycin SPECT/CT imaging for assessing cumulative apoptotic events in vivo. As shown in the presented images in **Fig 3D**, the normal control aortas in both axial and coronal views showed little uptake. Intense uptake was observed in both axial and coronal views in the model animals but was markedly reduced after 4 weeks of treatment with desipramine and atorvastatin. Gamma counting of the aortic segments further confirmed that the maximum uptake of ^99m^Tc-duramycin was observed in the Model group (2.52 ± 0.81%ID/kg), but was significantly lower in both desipramine (1.62 ± 0.55%ID/g; *P*<0.01) and atorvastatin (1.88 ± 0.44%ID/g; *P*<0.05) groups.

### Correlation between radiotracer uptake and morphology

**Fig 4:**
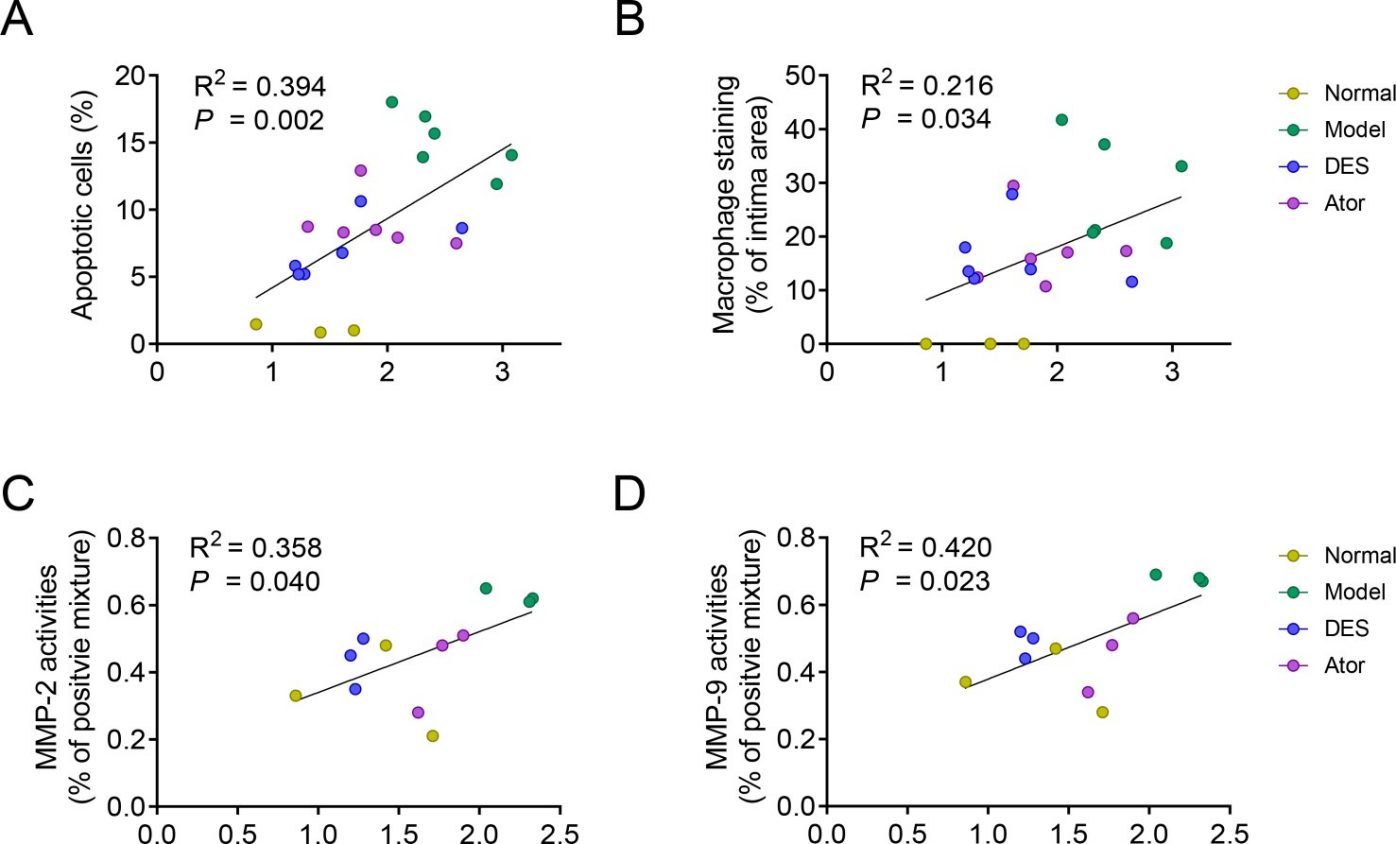
Correlations of quantitative radioactive uptake of ^99m^Tc-duramycin and plaques instability. **(A)** Correlations of quantitative radioactive uptake of ^99m^Tc-duramycin with apoptotic cells stained with TUNEL, **(B)** area stained by RAM-11 (expressed as macrophage percentage of total plaque area or total cell numbers), **(C, D)** and MMPs activities. n = 12 to 21.

It has been proven that macrophage infiltration and MMP secretion within atherosclerotic lesions were associated with plaque instability. **Fig 4A-4D** showed the correlation between ex vivo measurements of ^99m^Tc-duramycin uptake and morphological components. The respective piece-by-piece ID%/g correlated positively with proportion of apoptotic cell (%, *r*^2^ = 0.394; *P* = 0.002), immunopositive macrophages (%, *r*^2^ = 0.216; *P* = 0.034), MMP-2 activities (*r*^2^ = 0.358; *P* = 0.040) and MMP-9 activities (*r*^2^ = 0.420; *P* = 0.023).

## Discussion

In the present study, inhibition of ASM improved morphological stability associated with a reduction in the plaque content of macrophages, MMP-2, and MMP-9 expression, and attenuated lesional apoptosis in the aortic media of atherosclerotic rabbits, similar to atorvastatin treatment. Additionally, ^99m^Tc-duramycin imaging enabled non-invasive monitoring of atherosclerosis treatment and assessment of anti-apoptotic effects.

Lipid deposition is the initiating process for atherogenesis. Most existing therapies aim to lower cholesterol levels, but equally important circulating lipids, such as ceramides, have not been adequately addressed. Our study demonstrated that after 4-week of treatment with the ASM inhibitor, desipramine prevented ceramide accumulation in plasma and atherosclerotic plaques, and ameliorated abdominal aortic intima-media thickness in atherosclerotic rabbits. Notably, unlike atorvastatin treatment, no significant alterations in serum cholesterol were observed with desipramine treatment. These findings were consistent with previous data [29], suggesting that the mechanism linking desipramine suppressing atherogenesis is possibly involved in the vascular lipid uptake instead of involvement in circulating LDL particles [30].

The role of ASM in atherosclerosis in mouse studies is still debated and controversial. Previous studies demonstrated that deficiency of ASM reduced lipoprotein retention, foam cell formation, and attenuated lesion progression in ASM^-/-^/ApoE^-/-^ and ASM^-/-^/LDLR^-/-^ mice [15]. Unexpectedly, another study found that adenovirus-mediated expression and secretion of ASM into the circulation did not accelerate but rather decrease lesion formation in ApoE^-/-^ mice [31]. Since certain mechanisms of anti-atherogenic in mice may not be relevant to humans. In this study, we established a model of atherosclerosis in rabbits to investigate the effects of desipramine on atherosclerosis plaque stability. The so-called “vulnerable” plaques are characterized by large necrotic cores, focal thinning of the fibrous cap, apoptosis of intimal cells, and a high level of inflammatory cells, particularly macrophages which promote MMP levels and activity [32, 33]. Both MMP-2 and MMP-9 are associated with plaque vulnerability and cap rupture, predisposing to acute coronary syndrome [34, 35]. In the present study, desipramine treatment was found to attenuate macrophage accumulation and elevate SMC content compared to the model control rabbits. Here, atorvastatin-treated aorta tissue displayed a similar downward trend. These results are consistent with previous studies that reported changes in plaque composition with atorvastatin therapy [36, 37]. Moreover, both desipramine and atorvastatin were associated with reduced MMPs activities in plaques. Taken together, these results supported that desipramine potentially acts as a complement or replacement for statin therapy by facilitating to stabilize atherosclerotic plaques by reducing MMP expression

Apoptosis usually occurs in advanced atherosclerotic plaques and has the potential to contributes to plaque vulnerability. Our previous research reported that ASM mediated oxidized-LDL induced macrophages apoptosis via endoplasmic reticulum stress, and that inhibiting of ASM activity could attenuated apoptosis of human macrophages in vitro [26]. The present study further determined that the anti-apoptosis effect of inhibiting ASM could be recapitulated in animal experiments. The evidences from the TUNEL staining and duramycin imaging showed significantly reduced lesional apoptosis in the desipramine and atorvastatin groups, compared to the model control group. The reduction in apoptosis and lower duramycin uptake in atorvastatin group is understandable because of the decreased cholesterol level. Whereas the anti-apoptosis effect in desipramine group may be ascribed to a pleiotropic effect of lowering ceramide independent of cholesterol levels. Therefore, the present study identified the suppression of cell apoptosis in plaques as one of the underlying mechanisms behind the athero-protective function of desipramine.

^99m^Tc-duramycin is a phosphatidylethanolamine-binding agent with favorable pharmacokinetics and a safety profile for use in *in vivo* imaging [23]. Prior studies reported that ^99m^Tc-duramycin was specifically taken up by apoptotic cells in animal models of tumors [38], myocardial ischemia/reperfusion injury [39], and atherosclerosis [21]. More recently, Elvas et. al. showed the usefulness of ^99m^Tc-duramycin imaging for noninvasive, early prediction of response to tumor therapy [40]. Herein, by using a rabbit model of atherosclerosis, we verified the feasibility of using ^99m^Tc-duramycin to monitor atherosclerotic plaque apoptosis and stability. Importantly, ^99m^Tc-duramycin imaging positively correlated classical histological features of atherosclerosis plaques. For example, compared with the HFD group, the uptake of ^99m^Tc-duramycin was significantly lower in the groups treated with desipramine or atorvastatin, and this agreed with the histological analyses that showed fewer TUNEL-positive cells and less macrophage staining in these treatment groups. Given the positive correlation between noninvasive imaging and histological findings, the ^99m^Tc-duramycin imaging could be used for evaluating the treatment efficacy for atherosclerotic disease in the future.

This study has its limitations. Firstly, we used an animal model based on aortic balloon injury combined with a high-cholesterol diet without pharmacological triggering thrombosis. These plaques are characterized by significant vascular lipid and macrophage content deposition, but rarely a large lipid-rich necrotic core that differs from human plaques in many respects. Secondly, the ASM inhibitor desipramine also act as reuptake inhibitor for neurotransmitters in clinical practice, so effects of the neurotransmitters on atherosclerosis cannot be completely ruled out. Some researchers demonstrated that other neurotransmitter inhibitors, such as fluoxetine and sertraline, might exacerbate atherosclerosis [41, 42]. However. the present study found desipramine alleviated atherosclerosis, despite the potential exacerbating effects of neurotransmitters on atherosclerosis. Accordingly, desipramine’s anti-atherogenic effect appears to be mediated by the ceramide pathway rather than neurotransmitter inhibition, which requires further investigation. Thirdly, the positive control drug used in current experiment is atorvastatin, of which anti-atherogenic effect may exert both a lipid-lowering and ASM-suppressing influence [43]. Thus, the interference factor cannot be eliminated when compare the therapeutic effect of these two mechanisms. Fourthly, ^99m^Tc-duramycin uptake is associated with both apoptotic and necrotic cells, which may undermine desipramine’s antiapoptotic effects and explain the poor R^2^ value of the correlation with TUNEL. Thirdly, it is crucial to note that current clinical SPECT machines have a lower spatial resolution for imaging small objects when compared with pre-clinical SPECT machines. Therefore, using pre-clinical SPECT can be argued to be more practical to evaluate atherosclerotic plaque noninvasively for small animal models.

We suggest that ASM may be a potential target for the treatment of atherosclerosis. pharmacologically inhibiting ASM exert a stabilizing effect on the plaque in the rabbit balloon-injured arteries via reducing MMPs expression and cell apoptosis, which is summarized in graphic abstract. By using non-invasive imaging of ^99m^Tc-duramycin SPECT/CT, we could non-invasively observe the changes in cell apoptosis in atherosclerosis in vivo and assess the therapeutic effect of plaque stabilization.

## Supporting information

S1 Table Lipid profile of rabbits. Normal and atherosclerotic rabbits were treated with saline (Control), desipramine (DES), or atorvastatin (Ator), body weight, serum TG, TC, and LDL level were assessed. Data are expressed as Mean ± SD, n = 8; *, ** represent *P* <0.05, *P* <0.01 in comparison with Normal; #, ## represent *P* <0.05, *P* <0.01 in comparison with Model.
(DOCX)

S1 File (PDF)

S1 Raw images (PDF)

S1 Graphical abstract (TIF)
