# Supplementary material for: Desipramine enhances the stability of atherosclerotic plaque in rabbits monitored with molecular imaging
Source: PLoS One. 2023 Mar 30;18(3):e0283612. doi: 10.1371/journal.pone.0283612 (PMC10062573; doi:10.1371/journal.pone.0283612)
Supplement: S1 Table — Normal and atherosclerotic rabbits were treated with saline (Control), desipramine (DES), or atorvastatin (Ator), body weight, serum TG, TC, and LDL level were assessed. Data are expressed as Mean ± SD, n = 8; *, ** represent P <0.05, P <0.01 in comparison with Normal; #, ## represent P <0.05, P <0.01 in comparison with Model. (DOCX) [file pone.0283612.s001.docx]

**S1 Table. Lipid profile of rabbits**

|  | Normal | Model | DES | Ator |
| --- | --- | --- | --- | --- |
| Body weight (kg) | 3.759±0.244 | 4.171±0.296^*^ | 3.999±0.439 | 3.992±0.166 |
| TG (mmol/L) | 0.940±0.336 | 5.695±1.440^**^ | 5.491±1.578^**^ | 2.448±0.766^*, ##^ |
| TC (mmol/L) | 0.679±0.168 | 35.380±3.231^**^ | 34.03±1.95^**^ | 27.56±5.385^**, #^ |
| LDL (mmol/L) | 0.244±0.073 | 23±2.164^**^ | 22.2±8.056^**^ | 14.68±3.768^*, ##^ |

Normal and atherosclerotic rabbits were treated with saline (Control), desipramine (DES), or atorvastatin (Ator), body weight, serum TG, TC, and LDL level were assessed. Data are expressed as Mean ± SD, n = 8; *, ** represent P <0.05, P <0.01 in comparison with Normal; #, ## represent P <0.05, P <0.01 in comparison with Model.
