# Supplementary material for: Desipramine enhances the stability of atherosclerotic plaque in rabbits monitored with molecular imaging
Source: PLoS One. 2023 Mar 30;18(3):e0283612. doi: 10.1371/journal.pone.0283612 (PMC10062573; doi:10.1371/journal.pone.0283612)
Supplement: S1 File — (PDF) [file pone.0283612.s002.pdf]

Data in brief

The corresponding detail data and related blot pictures of figure in manuscript were attached as supporting information

Data set of Fig 1

Fig 1B. Plasma ASM activity (nmol/L)	Normal	Model	DES	Ator
	10.82	22.37	11.36	10.12
	10.28	16.61	15.78	17.41
	15.34	12.39	9.02	16.81
	13.49	20.93	12.94	11.09
	10.65	15.66	10.69	15.99
	11.52	16.10	15.60	11.27
	8.34	18.94	11.59	12.69
	6.48	23.46	8.72	19.80

Fig 1C. Plasma ceramide levels (nmol/L)	Normal	Model	DES	Ator
	1.30	5.90	1.60	3.58
	1.30	9.60	5.80	4.68
	0.50	11.50	4.80	4.36
	4.60	14.10	6.50	9.66
	0.20	6.00	6.30	6.44
	0.30	7.98	2.40	7.37
	6.40	15.45	5.90	8.97
	4.30	16.32	4.80	12.36

Fig 1D. ASM activity in aorta (nmmol/mg)	Normal	Model	DES	Ator
	0.80	2.54	0.94	1.62
	0.58	1.80	0.92	0.65
	0.69	1.92	0.55	1.76
	1.24	1.57	1.66	1.43
	0.75	1.56	1.01	1.15
	0.90	1.81	0.81	1.06
	0.87	1.88	1.07	0.84
	0.66	2.59	1.73	1.10

Fig 1E. Ceramide levels in aorta (nmol/mg)	Normal	Model	DES	Ator
	1.47	4.06	2.7	2.37
	2.35	3	1.5	4.19
	2.26	3.79	1.84	1.34
	2.58	4.48	2.03	4.02
	0.84	4.84	1.45	1.35
	0.95	4.12	1.92	2.61
	2.27	3.05	1.97	2.06
	2.47	3.37	4.37	2.36

Effects of desipramine and atorvastatin on lipid profiles. New Zealand white rabbits were randomly divided into four groups: Rabbits were fed on normal chow diet served as normal controls (Normal); Abdominal aorta balloon injury surgery was performed on high-cholesterol diet feeding rabbits after 2 weeks, then normal saline (Model), desipramine (DES), and atorvastatin (Ator) were administrated by gavage from 8th to 12th week. Finally, blood was obtained, and ASM activities and ceramide levels in plasma were determined by UPLC; ASM activities and ceramide levels in aorta tissue were assessed by UPLC.

Data set of Fig 2

Fig 2B. Intima / Media area (%)	Normal	Model	DES	Ator
	0.00	2.81	3.72	2.32
	0.00	2.04	1.46	2.15
	0.00	3.93	2.22	1.26
	0.00	5.54	2.59	1.80
	0.00	4.00	2.41	2.51
	0.00	3.56	2.64	1.74

Fig 2C. SMC staining (% of intima area)	Normal	Model	DES	Ator
	0.00	11.79	18.05	20.99
	0.00	14.82	26.12	20.94
	0.00	11.72	15.77	16.76
	0.00	10.50	26.05	17.47
	0.00	16.00	25.50	22.52
	0.00	14.53	22.85	17.97

Fig 2D. Macrophage staining (% of intima area)	Normal	Model	DES	Ator
	0.00	21.17	12.16	15.85
	0.00	41.73	17.99	10.74
	0.00	20.71	13.54	29.45
	0.00	18.78	13.89	12.38
	0.00	33.09	11.60	17.29
	0.00	37.13	27.87	17.04

Desipramine and atorvastatin enhances atherosclerotic plaque stability. Normal and atherosclerotic rabbits were treated with saline (Model), desipramine (DES), or atorvastatin (Ator), representative histological and Immunohistological staining of abdominal aortic plaque components including alpha smooth muscle actin (α-SMA) staining of SMCs, and RAM-11 staining of macrophages. The ratio of intima/media area in each rabbit was analyzed; Quantitative analysis of SMCs and macrophages in 4 groups were performed.

Data set of Fig 3

Fig 3A. MMPs positive area (%)	Normal	Model	DES	Ator
MMP-2	0	39.93	18.55	21.76
	0	21.51	34.01	17.3
	0	49.35	32.86	37.95
	0	25.86	15.78	20.1
	0	44.43	24.78	30.44
	0	36.18	22.6	17.73
MMP-9	0	39.93	18.55	21.76
	0	21.51	34.01	17.3
	0	49.35	32.86	37.95
	0	25.86	15.78	20.1
	0	44.43	24.78	30.44
	0	36.18	22.6	17.73

Fig 3B. MMPs activities (% of positvie mixture)	Normal	Model	DES	Ator

MMP-2	0.33	0.62	0.50	0.48
	0.48	0.65	0.45	0.51
	0.21	0.61	0.35	0.28
MMP-9	0.37	0.67	0.50	0.48
	0.47	0.69	0.52	0.56
	0.28	0.68	0.44	0.34

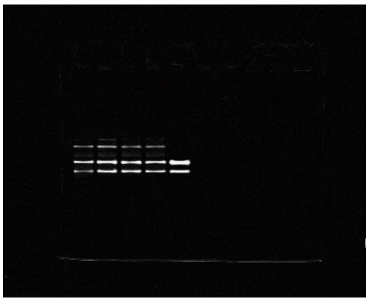
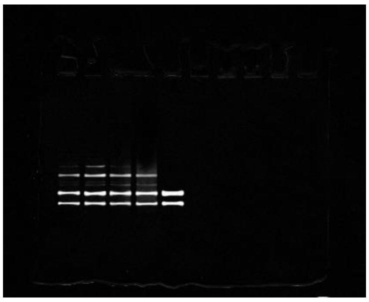
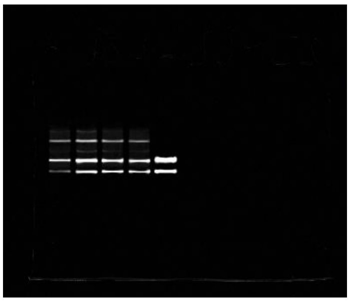

Fig 3C. Apoptotic cells (% of lesion area)	Normal	Model	DES	Ator
	1.47	16.95	5.21	12.92
	0.85	18.01	5.81	8.50
	1.01	13.92	5.19	8.31
	1.24	11.92	10.62	8.73
	1.20	14.06	8.62	7.50
	0.65	15.67	6.79	7.92

Fig 3D. 99mTc-duromycin (%ID/g)	Normal	Model	DES	Ator
	0.86	2.33	1.28	1.77
	1.42	2.04	1.20	1.90
	1.71	2.31	1.23	1.62
		2.95	1.77	1.31
		3.08	2.65	2.60
		2.41	1.61	2.09

Desipramine and atorvastatin inhibit MMPs and apoptosis in atherosclerotic plaques. Zymographic analysis of MMP-2 and MMP-9 activities, the densitometry of each band was determined by ImageJ software and calculated as the percentage of the positive control (PC) on each gel. Quantification of TUNEL-positive (green) cells in aortic atherosclerotic plaques were analyzed. *In vivo* imaging of abdominal aorta (arrowheads) with ^{99m}Tc-duramycin SPECT/CT and measurement of radioactive ^{99m}Tc-duramycin uptake in aortic.

Data set of Fig 4

	Normal	Model	DES	Ator
duro	0.86	2.33	1.28	1.77
	1.42	2.04	1.20	1.90
	1.71	2.31	1.23	1.62
		2.95	1.77	1.31
		3.08	2.65	2.60
		2.41	1.61	2.09

	Normal	Model	DES	Ator
tunel	1.47	16.95	5.21	12.92
	0.85	18.01	5.81	8.50
	1.01	13.92	5.19	8.31
		11.92	10.62	8.73
		14.06	8.62	7.50
		15.67	6.79	7.92

	Normal	Model	DES	Ator
macrophage	0.00	21.17	12.16	15.85
	0.00	41.73	17.99	10.74
	0.00	20.71	13.54	29.45
		18.78	13.89	12.38
		33.09	11.60	17.29
		37.13	27.87	17.04

MMPs activities (% of positvie mixture)	Normal	Model	DES	Ator
MMP-2	0.33	0.62	0.50	0.48
	0.48	0.65	0.45	0.51
	0.21	0.61	0.35	0.28
MMP-9	0.37	0.67	0.50	0.48
	0.47	0.69	0.52	0.56
	0.28	0.68	0.44	0.34

Correlations of quantitative radioactive uptake of ^{99m}Tc-duramycin and plaques instability. Correlations of quantitative radioactive uptake of ^{99m}Tc-duramycin with apoptotic cells stained with TUNEL, area stained by RAM-11 (expressed as macrophage percentage of total plaque area or total cell numbers), and MMPs activities were analyzed. Each position represents the sample from a single rabbit.
