# Supplementary figures and images for: Desipramine enhances the stability of atherosclerotic plaque in rabbits monitored with molecular imaging
Source: PLoS One. 2023 Mar 30;18(3):e0283612. doi: 10.1371/journal.pone.0283612 (PMC10062573; doi:10.1371/journal.pone.0283612)

| Fig 3B. MMPs activities (% of positive mixture) | Normal | Model | DES  | Ator |
|-------------------------------------------------|--------|-------|------|------|
| MMP-2                                           | 0.33   | 0.62  | 0.50 | 0.48 |
|                                                 | 0.48   | 0.65  | 0.45 | 0.51 |
|                                                 | 0.21   | 0.61  | 0.35 | 0.28 |
| MMP-9                                           | 0.37   | 0.67  | 0.50 | 0.48 |
|                                                 | 0.47   | 0.69  | 0.52 | 0.56 |
|                                                 | 0.28   | 0.68  | 0.44 | 0.34 |

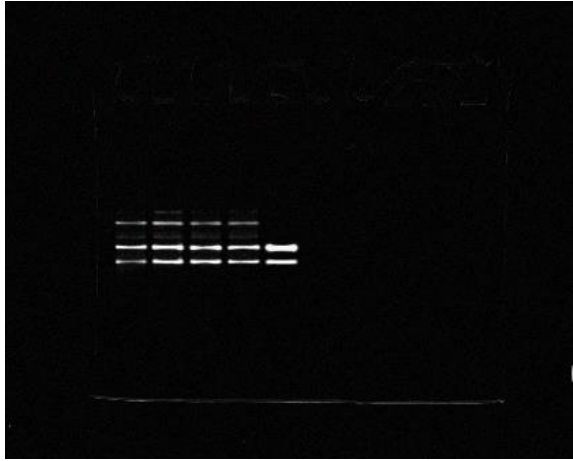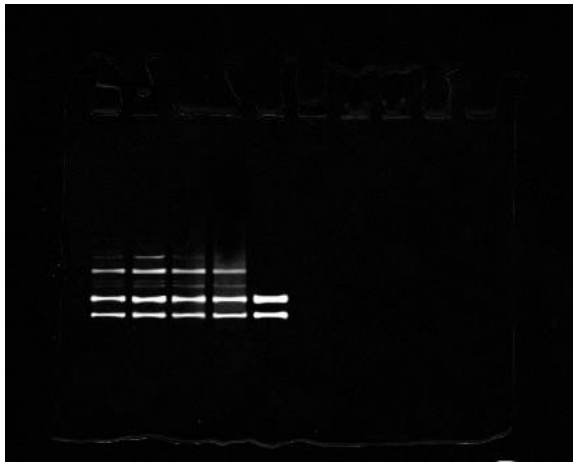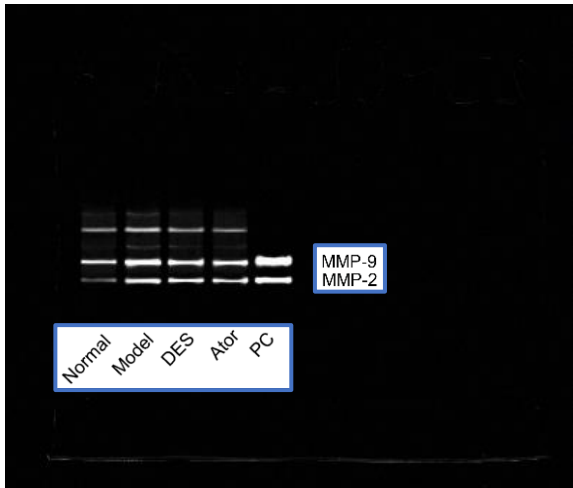

Supplement: S1 Raw images — (PDF) [file pone.0283612.s003.pdf]

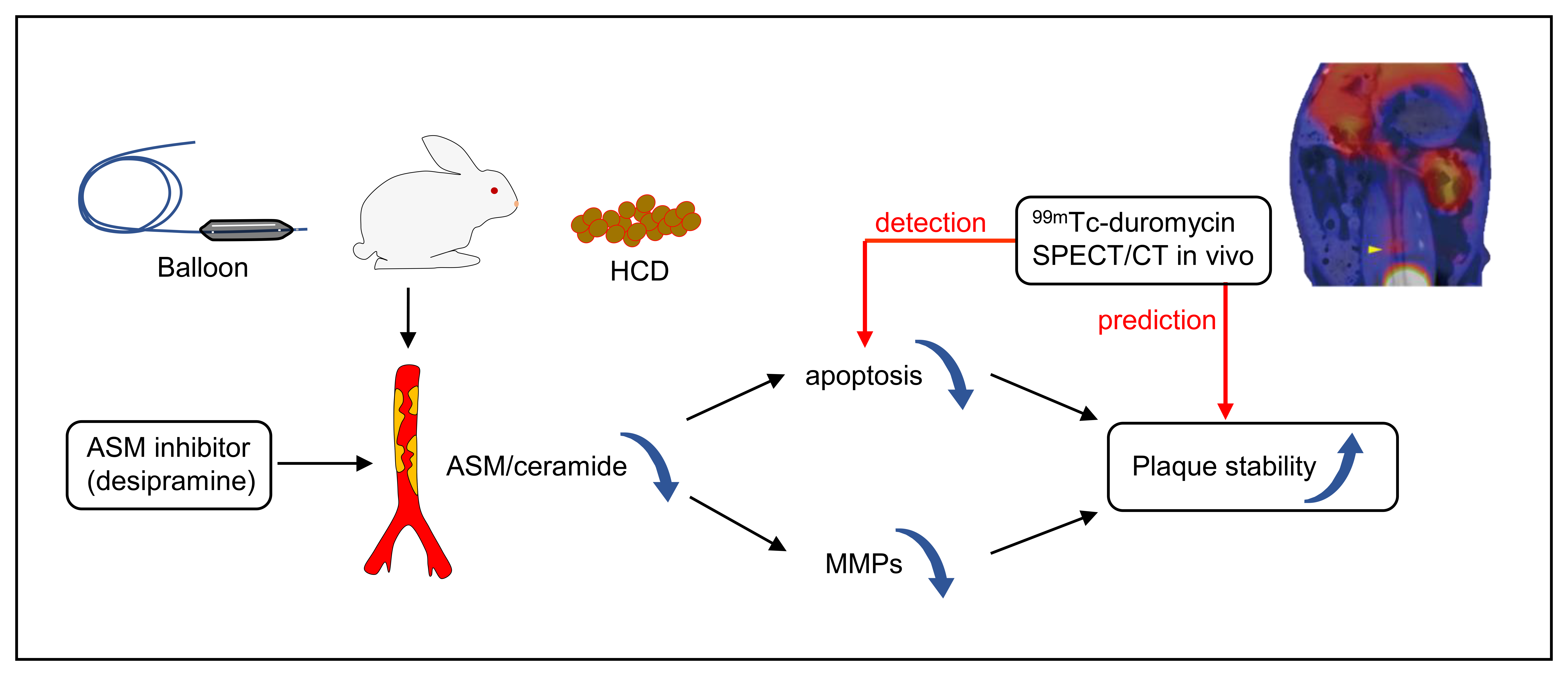

Supplement: S1 Graphical abstract — (TIF) [file pone.0283612.s004.tif]
